# Uterine torsion with scoliosis

**DOI:** 10.1002/ccr3.4818

**Published:** 2021-09-13

**Authors:** Kyosuke Kamijo, Fumika Kubota, Ikuo Yoshioka

**Affiliations:** ^1^ Department of Obstetrics and Gynecology Nagano Prefectural Kiso Hospital Kiso Japan

**Keywords:** abdominal pain, scoliosis, uterine torsion

## Abstract

Clinicians should consider uterine torsion as a differential diagnosis for acute abdominal pain in women with scoliosis. Scoliosis may have led to an abnormal pelvic structure, making it easier for pelvic organs to twist.

## Graphical abstract

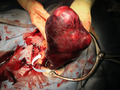

## 1 QUESTION

What is the differential diagnosis of abdominal pain with scoliosis?

## 2 ANSWER

A differential diagnosis of sudden abdominal pain with scoliosis indicates uterine torsion, a rare condition in clinical practice.

## 3 MANUSCRIPT

**FIGURE 1:**
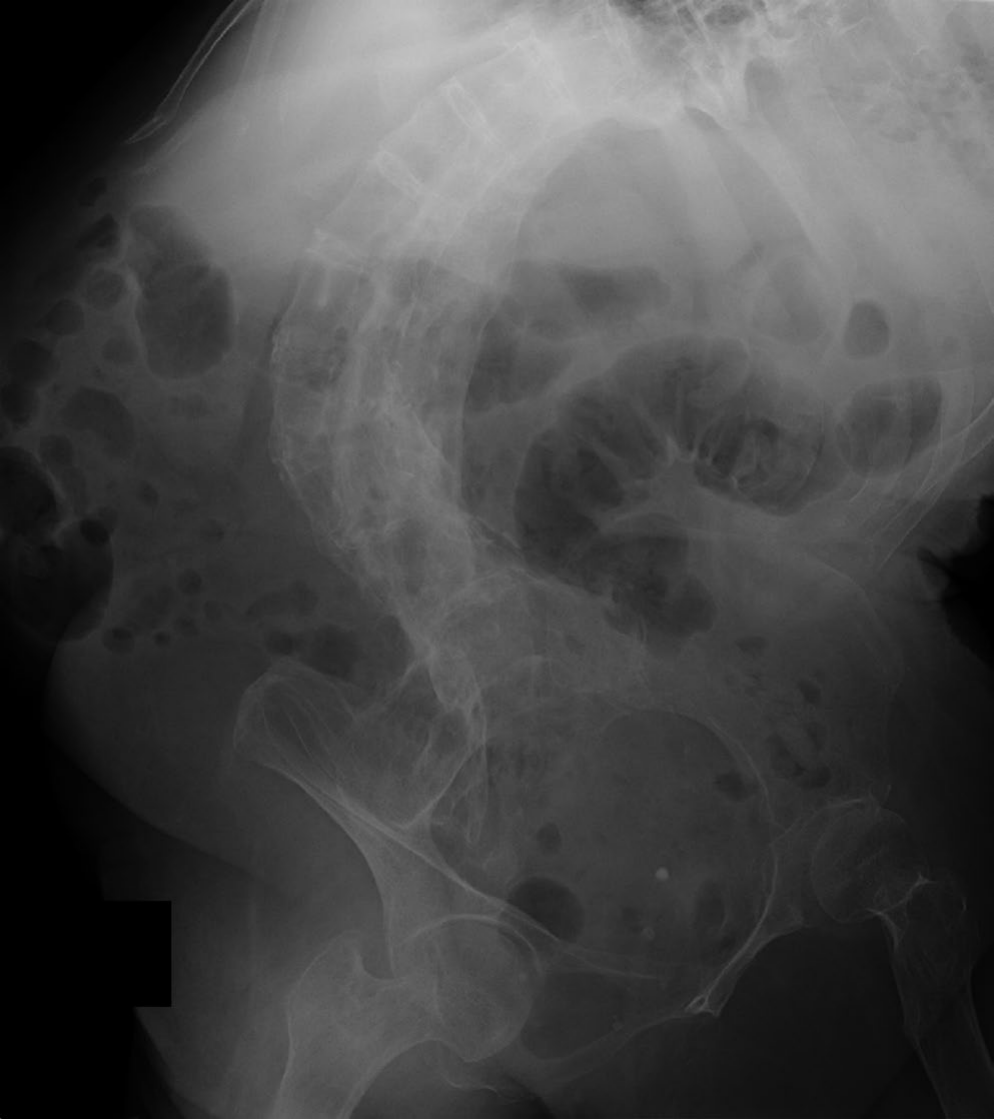
X‐ray of the abdomen indicates severe scoliosis

**FIGURE 2:**
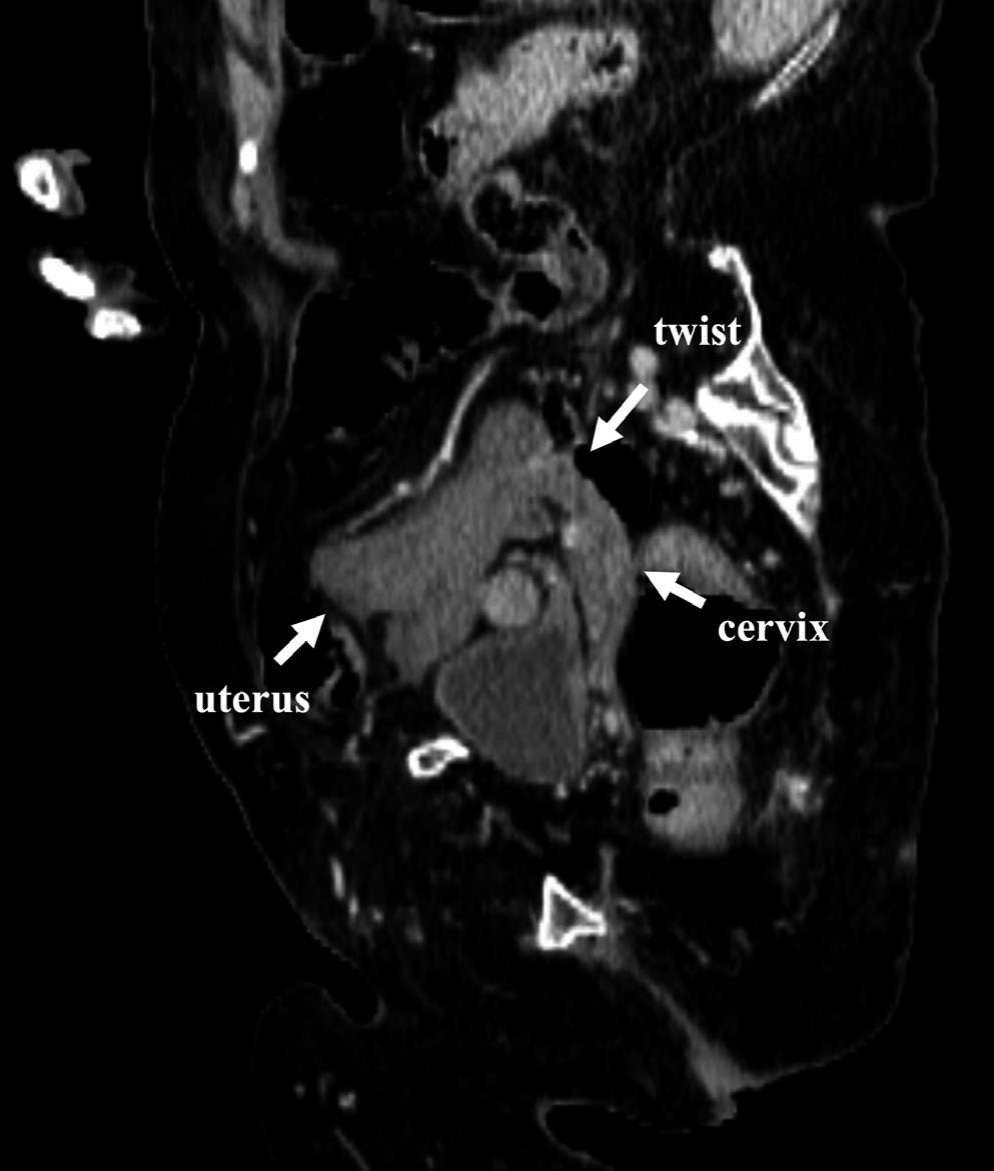
CT of the abdomen and pelvis shows torsion of the cervix (arrow)

A 73‐year‐old woman with a history of infantile paralysis and scoliosis presented with sudden abdominal pain. Physical examination revealed a palpable mass in the lower abdomen. X‐ray revealed severe scoliosis (Figure 1). Contrast‐enhanced CT showed a uterine mass (20 cm) across its widest dimension without enhancement effect and cervical torsion (Figure 2). We observed a 5‐cm‐sized left ovarian tumor and a 10‐cm‐sized right ovarian tumor.

**FIGURE 3:**
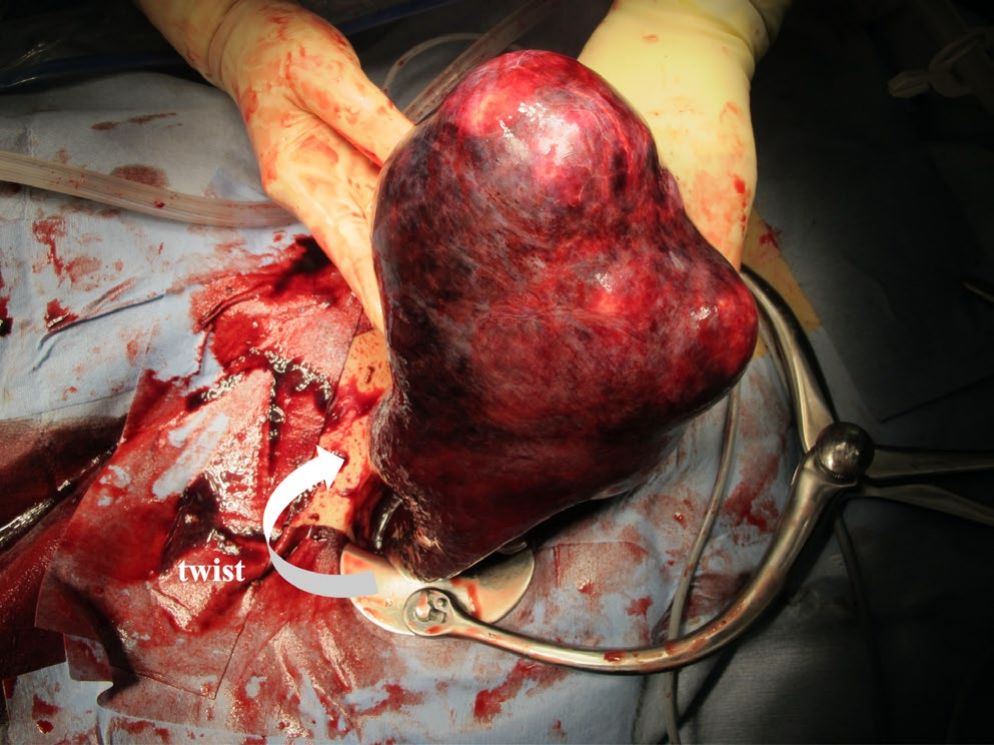
Laparotomy reveals the mostly necrotic uterus twisted 180º

Based on the preoperative diagnosis of either side of ovarian torsion, total hysterectomy and bilateral salpingo‐oophorectomy were performed. Intraoperatively, the uterus appeared mostly necrotic and twisted by 180º (Figure 3). The uterine and bilateral ovarian tumors were in the right pelvis at the abnormal deep portion where the spine had curved. Histopathological analysis revealed uterine leiomyomas with congestive necrosis and bilateral ovarian serous cystadenoma. Her postoperative course was uneventful.

Uterine torsion, defined as >45º twisting of the uterus on its long axis, is rare.^1^ Cases of volvulus with scoliosis and uterine torsion with ovarian cyst have been reported.^2^, ^3^ Our case may illustrate the association between uterine torsion and scoliosis. Scoliosis may have led to an abnormal pelvic structure, making it easier for the massive uterus and ovarian tumors to twist.

## ACKNOWLEDGMENTS

The authors would like to thank the patient for giving consent.

## CONFLICT OF INTEREST

None declared.

## AUTHOR CONTRIBUTIONS

KK provided case information, references, and wrote the manuscript. FK and IY provided case information and clinical images.

## ETHICAL APPROVAL

Standard ethical approval was obtained.

## CONSENT

Published with written consent of the patient.

## Data Availability

The data that support the findings of this study are available from the corresponding author upon reasonable request.
